# Expression Analysis of Selected miRNAs in COVID-19 Patients

**DOI:** 10.3390/microorganisms14092064

**Published:** 2026-09-16

**Authors:** Gökçe Kader Arslan, Mehmet Özdemir

**Affiliations:** 1 Medical Microbiology Department, Patnos State Hospital, 04500 Ağrı, Türkiye; 2 Division of Medical Virology, Medical Microbiology Department, Faculty of Medicine, Necmettin Erbakan University, 42080 Konya, Türkiye; mehmetozdem@yahoo.com

**Keywords:** COVID-19, miR-1307-3p, miR-26a-5p, miR-21, let-7b-3p

## Abstract

Modulations in microRNA (miRNA) expression can contribute to disease pathogenesis and biological processes by regulating target proteins. We aimed to compare serum miRNA levels (miR-19a-3p, miR-19b-3p, miR-21, miR-26a-5p, miR-146a, let-7b-3p, miR-155, miR-16, miR-590, miR-1307-3p) between COVID-19 patients and healthy controls to investigate their clinical significance and contribution to disease pathogenesis. Blood samples were obtained from 70 patients diagnosed with COVID-19 and from 30 healthy individuals as a control group for comparison. The patients were stratified into three categories based on disease severity: mild, moderate, and severe. miRNA expression levels were quantified via real-time PCR, with relative quantification performed using the Livak ΔΔCT method by calculating the 2^(−ΔΔCT)^ fold change values. In patients with COVID-19, the expression levels of miR-1307-3p, miR-146, miR-26a-5p, miR-21, miR-19b-3p, and let-7b-3p demonstrated statistically significant differences (*p* < 0.05). A non-significant downward trend of miR-16 was observed, with miRNA expression profiles exhibiting variability across the different patient groups. Considering host–virus interactions, we posit that miRNAs hold significant potential both as novel therapeutic targets and as pivotal regulators in deciphering the intricate pathogenesis of COVID-19. Comprehensive elucidation of disease pathogenesis requires the synergistic integration of in vivo and in vitro experimental approaches in conjunction with rigorous clinical investigations.

## 1. Introduction

SARS-CoV-2 is the etiological agent responsible for COVID-19, a pandemic characterized by substantial morbidity and mortality rates. The virus is implicated in the pathogenesis of a spectrum of severe complications, including cardiac manifestations such as arrhythmias, acute coronary syndrome, heart failure, myocardial injury, and venous thromboembolism, alongside acute respiratory distress syndrome, multi-organ dysfunction syndrome, and various neurological sequelae [1,2].

MicroRNAs (miRNAs) are short, non-coding RNA molecules, typically 19–22 nucleotides in length, that intricately modulate a wide array of physiological processes through the sequence-specific targeting of messenger RNAs, leading to translational repression and/or mRNA degradation. miRNAs serve pivotal functions in modulating inflammatory responses—either by attenuation or activation—and are integral to the maintenance of immunological homeostasis [3]. miRNAs are ubiquitously present across all bodily fluids, including blood, exhibiting remarkable stability against degradation. Moreover, fluid-specific miRNA profiles are linked to distinct functional roles pertinent to their tissue microenvironment [4,5]. They play a pivotal role in a range of biological processes, including antiviral defense mechanisms and the regulation of host inflammatory signaling. Circulating miRNAs serve as key modulators in host–pathogen interactions, mediating host immune responses directed against the invading pathogens [6].

Host-derived miRNAs may be exploited by viruses to facilitate their own survival, or alternatively, they can initiate host immune responses that activate defensive pathways to counteract the infection. As a result of infection, their expression levels may be modulated—either upregulated or downregulated—due to their functional involvement in mediating viral entry and replication into host cells [7]. A substantial number of human miRNAs have demonstrated the potential to interact directly with the coronavirus’s genome. The interaction between miRNAs and their target biomolecules is thought to play a significant role in the COVID-19 progression [8]. Specific miRNA concentrations in body fluids hold the potential to serve as biomarkers for the detection and monitoring of various pathophysiological conditions [4].

The miRNA panel for this study (miR-1307-3p, miR-155, miR-146a, miR-21, miR-26a-5p, miR-19a/b-3p, miR-16, miR-590, and let-7b-3p) was strategically curated to encompass the multi-faceted nature of SARS-CoV-2 pathogenesis. miR-155 and miR-146a were selected based on prior reports identifying them as regulators associated with cytokine-storm-related signaling pathways [9,10]. miR-1307-3p was included to investigate viral–host interactions, given its predicted sequence complementarity to regions of the SARS-CoV-2 genome and its potential role in modulating ACE2 receptor expression [11]. miR-26a-5p was selected due to its reported cardiovascular effects [12]. Furthermore, the inclusion of miR-590, miR-21 and miR-19a/b-3p may provide an insight into COVID-19-related immune modulation [13,14,15]. Finally, miR-16 and let-7b-3p were selected because of their well-established involvement in cellular apoptosis and systemic antiviral defense mechanisms [16,17].

While several prior studies have profiled multiple circulating miRNAs in COVID-19 patients [18,19], the specific combination of markers evaluated here—spanning cytokine-storm regulators, a putative viral-entry modulator, cardiovascular-associated miRNAs, and apoptosis/antiviral-defense miRNAs within a single targeted qPCR panel—has not, to our knowledge, been jointly assessed in a Turkish cohort. Moreover, this study explicitly and separately tests whether these markers distinguish disease presence (patients vs. controls) from disease severity (mild vs. moderate vs. severe), a distinction not always addressed in prior reports [18,19].

Host–virus interactions during SARS-CoV-2 infection can lead to changes in the miRNA expression profile, which regulates various steps of the innate and acquired immune response [7,8]. Circulating miRNAs stand out as potential diagnostic and prognostic biomarkers due to their non-invasive sampling capabilities [4,20]. This study investigated whether serum expression levels of ten miRNAs implicated in COVID-19 pathogenesis (i) differ between COVID-19 patients and healthy controls and (ii) differ across mild, moderate, and severe disease subgroups.

## 2. Materials and Methods

This study encompassed seventy patients diagnosed with COVID-19 alongside thirty healthy controls. Detection of SARS-CoV-2 RNA was performed utilizing Bio-Speedy COVID-19 RT qPCR kits (Bioeksen, Istanbul, Türkiye) on a Rotor-Gene Q thermal cycler (Qiagen, Hilden, Germany). Amplification curves exhibiting a cycle threshold (Ct) value below 38 with a sigmoidal profile were deemed positive. At initial hospital presentation, all patients underwent simultaneous collection of respiratory specimens for SARS-CoV-2 RT-qPCR, chest imaging, peripheral oxygen saturation (SpO_2_) measurement, and venous blood sampling for serum miRNA quantification and laboratory parameters, ensuring that miRNA sampling occurred at a comparable point in the clinical course across all severity groups. Clinical presentations of the patients were subsequently stratified into mild, moderate, and severe categories in accordance with WHO guidelines: mild disease was defined as symptomatic infection without clinical or radiological evidence of pneumonia or hypoxia; moderate disease as clinical signs of pneumonia (fever, cough, dyspnea, tachypnea) without signs of severe pneumonia, with SpO_2_ ≥ 90% on room air; and severe disease as clinical signs of pneumonia plus at least one of the following criteria: respiratory rate > 30 breaths/min, severe respiratory distress, or SpO_2_ < 90% on room air, with the most critically ill patients within this group requiring intensive care unit admission and, in some cases, invasive mechanical ventilation. Patients with mild disease were managed on an outpatient basis, whereas those with moderate and severe disease were hospitalized (median length of stay 7.76 ± 1.45 and 20.84 ± 5.36 days, respectively); this classification was further supported by a strong correlation with the respiratory support level required (ρ = 0.957, *p* < 0.001) and with the length of hospital stay (*p* < 0.001). Healthy control participants were required to have no history of SARS-CoV-2 infection or prior COVID-19 diagnosis, no active infection within the preceding month, and no chronic diseases; individuals not meeting these criteria were excluded from the control group.

Demographic data were retrieved from records in the hospital information system. The D-dimer assay kit was changed during the study period; all D-dimer values were retrospectively harmonized to a single unit (ng/mL FEU) prior to statistical analysis.

Serum samples were stored at −80 °C until the day of the study to enable the quantification of ten miRNAs (miR-1307, miR-155, miR-146, miR-26a-5p, miR-21, miR-19a-3p, miR-19b-3p, miR-16, miR-590, let-7b-3p) using real-time PCR.

miRNA extraction was performed using the SanPrep Column MicroRNA Mini-Prep Kit (Bio Basic Inc., Markham, ON, Canada). A total of 500 μL of serum was carefully transferred into RNase-free Eppendorf tubes, and miRNA isolation was conducted in accordance with the manufacturer’s protocol. Complementary DNA (cDNA) synthesis from miRNA was executed utilizing the miRNA All-In-One cDNA Synthesis Kit (Applied Biological Materials (abm) Inc., Richmond, BC, Canada). Subsequent quantification of miRNA expression levels was carried out employing the real-time PCR kit (Applied Biological Materials Inc., Richmond, BC, Canada) in accordance with standardized protocols. During the synthesis of cDNA from miRNAs, each sample’s reaction mixture was meticulously prepared in precise proportions as stipulated by the manufacturer’s protocol, with a total reaction volume of 20 μL. The mixture was then subjected to a controlled thermal cycling regimen using a Bio-Rad thermal cycler (Bio-Rad Laboratories, Inc., İstanbul, Turkey), entailing incubation at 37 °C for 30 min, followed by 15 min at 50 °C. The reaction was subsequently halted by heating at 85 °C for 5 min, thereby completing cDNA synthesis. Prior to proceeding with PCR amplification, the concentration of the synthesized cDNA was quantified via Nanodrop spectrophotometry (Thermo Fisher Scientific, Waltham, MA, USA); all samples were standardized to 100 ng. miRNA quantification was performed using a poly(A) tailing-based universal reverse transcription approach (miRNA All-In-One cDNA Synthesis Kit, Applied Biological Materials Inc., Richmond, BC, Canada) rather than stem-loop RT-PCR. This method was selected because it enables simultaneous reverse transcription of all target miRNAs and the reference gene in a single reaction using a universal primer, improving throughput and reducing input RNA requirements compared with the miRNA-specific stem-loop primers required for each individual target in stem-loop RT-PCR. Primer sequences are proprietary to the manufacturer (Applied Biological Materials Inc., Richmond, BC, Canada) and are not publicly disclosed.

To facilitate amplification of the cDNAs for the reference gene and to label the relevant regions, BlasTaq^TM^ 2× qPCR Master Mix was prepared.

The prepared reference gene (U6 snRNA) real-time PCR mixes and target gene real-time PCR mixes were combined with the appropriate cDNA samples in 96-well plates compatible with the LightCycler^®^ 96 system, followed by application of the thermal cycling protocol. The real-time PCR process was conducted using the LightCycler^®^ 96 thermal cycler (Roche Diagnostics, Rotkreuz, Switzerland). Finally, the cycle threshold (Ct) values were recorded.

Descriptive statistics were expressed as means ± standard deviations, while categorical variables were summarized using frequencies and percentages. Relative expression levels were determined employing Livak’s ΔΔCT method, calculating the corresponding 2^(−ΔΔCT)^ values [21]. Prior to hypothesis testing, the normality of ΔCt values within each group was assessed using the Shapiro–Wilk test, and the homogeneity of variances across groups was evaluated using Levene’s test. For each miRNA, one-way ANOVA followed by Tukey’s HSD post hoc test was applied when ΔCt values were normally distributed within all groups and variances were homogeneous (*p* ≥ 0.05 for both Shapiro–Wilk and Levene’s test). When these assumptions were violated, the Kruskal–Wallis rank sum test was used, followed by Dunn’s post hoc test with Benjamini–Hochberg correction for pairwise comparisons. For categorical variables, Pearson’s Chi-squared test was used when expected cell counts were ≥5 in all cells; otherwise, Fisher’s exact test was applied. The specific test used for each variable is indicated in the corresponding table footnotes. Furthermore, due to significant differences in age and gender distribution among disease severity groups, an ANCOVA (age-adjusted group comparison) including age as a covariate was applied for each miRNA. Because samples from each disease-severity group were processed in contiguous groups during data collection, we performed a post hoc sensitivity analysis to evaluate the potential influence of U6 snRNA variability on the observed between-group differences. This evaluation comprised three steps: (i) comparison of U6 Ct values across disease-severity groups using the Kruskal–Wallis test, with the coefficient of variation reported as a complementary descriptive measure of within-group variability; (ii) correlation analysis between U6 Ct values and raw (non-normalized) target miRNA Ct values to evaluate the extent of shared variation between the endogenous reference and target assays; and (iii) rank-based covariate adjustment for U6 Ct, applied separately to each of the ten miRNAs to determine whether the group effects observed in the primary analyses remained robust after accounting for variation in U6 Ct. All analyses were performed using R software version 4.2.2, with a *p* < 0.05 considered statistically significant.

To further evaluate potential non-linear effects of age on miRNA expression, a quadratic age term was added to the covariate-adjustment model for each miRNA. We additionally assessed the feasibility of an age-matched subgroup analysis; however, given the minimal overlap in age distribution between the control group (range 25–53 years) and the moderate/severe patient groups (range 49–88 years), a matched-subgroup analysis was determined to be statistically underpowered and was not pursued.

Potential target genes of the selected miRNAs (miR-1307, miR-155, miR-146a, miR-26a-5p, miR-21, miR-19a-3p, miR-19b-3p, miR-16, miR-590, and let-7b-3p) were predicted using the TargetScan (www.targetscan.org, accessed on 9 April 2026), miRDB (www.mirdb.org, accessed on 9 April 2026), and miRTarBase (https://mirtarbase.cuhk.edu.cn, accessed on 9 April 2026) databases. The overlapping genes were subjected to protein–protein interaction (PPI) network analysis using the STRING database (version 11.5). *Homo sapiens* was selected as the reference organism and an interaction confidence score of ≥0.7 was applied. Functional enrichment analysis, including Gene Ontology and KEGG pathway analysis, was performed using Enrichr (https://maayanlab.cloud/Enrichr/, accessed on 9 April 2026) to identify inflammation-related biological processes and signaling pathways. Hub genes were determined based on node degree within the PPI network.

## 3. Results

The study group comprised 70 individuals diagnosed with COVID-19 via qRT-PCR and 30 healthy controls for control group. All 70 patients included in the study had RT-qPCR-confirmed SARS-CoV-2 infection (Ct value < 38 with a sigmoidal amplification curve), and all 30 individuals in the control group tested negative for SARS-CoV-2 RNA by the same assay. Among the patient group, 41 (58.57%) were male and 29 (41.43%) were female. The control group consisted of an equal distribution of males and females (15 each, 50%). The mean age of the control participants was 36.03 ± 8.89 years. Compared to the type of O2 support (nasal/high-flow/intubation), there was a very strong consistency of rho = 0.957 (*p* < 0.001). Patients with mild disease were managed on an outpatient basis and therefore had no hospital admission. Among hospitalized patients, length of stay was significantly longer in the severe group (20.84 ± 5.36 days) compared with the moderate group (7.76 ± 1.45 days; Welch’s *t*-test, *p* < 0.001), providing further support for the validity of the severity classification. Overall, 14 of 70 patients (20.0%) died during hospitalization, all within the severe subgroup (56.0% mortality in this group); no deaths occurred among mild or moderate cases. This confirmed the clinical severity of the patients. Detailed demographic and biochemical characteristics of the patient groups are presented in Table 1 and Table 2.

U6 small nuclear RNA (U6 snRNA) was used as the endogenous reference gene for the normalization of miRNA expression data (ΔCt calculation). Although miR-16 is also commonly used as a reference gene in circulating miRNA studies, it was not suitable here because it was itself one of the ten target miRNAs under investigation; using it simultaneously as a normalizer and as an analyte of interest would introduce circularity into the analysis. U6 was selected at the time of study design based on its established and widespread use in the miRNA normalization literature [22]. We note that this choice is further corroborated by a subsequently published, independent study demonstrating U6 stability specifically in COVID-19 patient–control comparisons [23], lending additional post hoc support to our original methodological decision. Subsequently, the expression profiles of ten miRNAs were analyzed across the COVID-19 patients and the control group, as shown in Table 3. Among the ten miRNAs, only miR-19a-3p showed a nominally significant difference among the mild, moderate, and severe patient groups (raw *p* = 0.032); however, this association did not remain statistically significant after correction for multiple comparisons (Bonferroni *p* = 0.325; Benjamini–Hochberg *p* = 0.317). Accordingly, no miRNA in this panel reliably distinguished disease severity subgroups after appropriate statistical correction.

Although miR-16 did not differ significantly between the control and pooled COVID-19 groups (*p* = 0.063), a post hoc pairwise comparison showed a significant difference between the control and severe groups (Benjamini–Hochberg-corrected *p* = 0.0024), suggesting that differences in miR-16 expression may be more apparent in severe disease than in the overall patient cohort.

Furthermore, statistical analyses of ΔCT values comparing the control group with each patient subgroup were provided in Table 4. Age-adjusted analysis of covariance (ANCOVA) was performed for each miRNA to assess whether group differences persisted after considering age. For miR-26a-5p, miR-21, miR-19a-3p, 19b-3p, and let-7b-3p, group effects remained statistically significant after age adjustment (all *p* < 0.05), exhibiting the idea that these associations cannot be fully explained by age. For miR-1307-3p and miR-146a, statistical significance decreased but was maintained after adjustment (*p* = 0.047 and *p* = 0.031, respectively), suggesting that these findings require more cautious interpretation.

Inclusion of a quadratic age term did not reach statistical significance for any of the ten miRNAs (all *p* > 0.18), and group-effect *p*-values remained materially unchanged after adding this term (Supplementary Table S1), providing no evidence of non-linear age effects influencing the observed associations.

**Figure 1:**
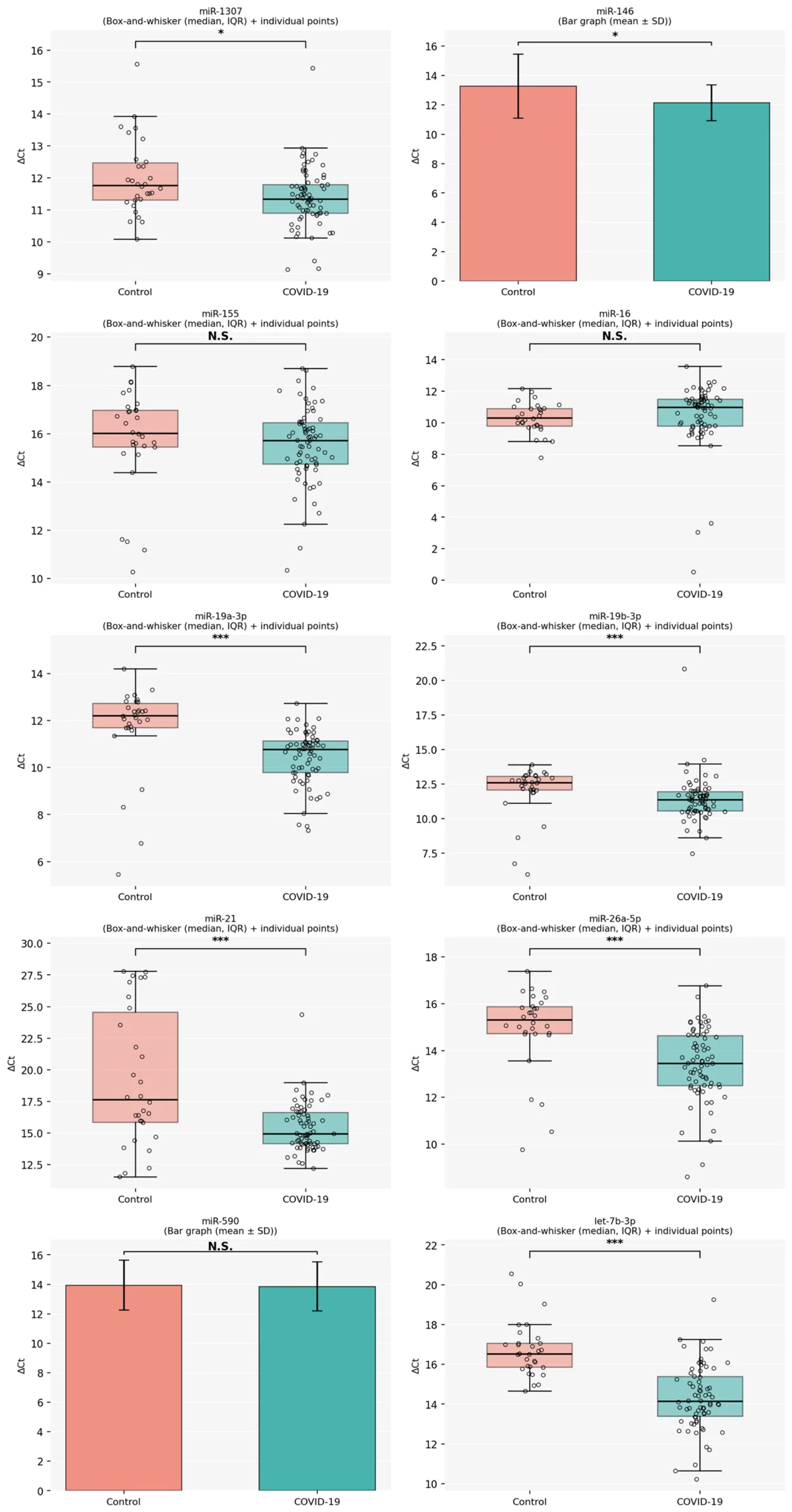
Relative expression levels of selected miRNAs in control and patient groups. N.S.: not significant; * *p* < 0.05; *** *p* < 0.001.

Subsequently, relative expression levels were calculated using the 2^(−ΔΔCT)^ method. Based on these values, differences in miRNA expression levels between the patient and control groups were identified, and the resulting expression profiles were compared. The serum expression levels of miR-1307-3p (1.5-fold, *p* < 0.01), miR-155 (1.18-fold, *p* 0.146), miR-146 (2.2-fold, *p* < 0.01), miR-26a-5p (2.85-fold, *p* < 0.001), miR-21 (14.6-fold, *p* < 0.001), miR-19a-3p (2.44-fold, *p* < 0.001), miR-19b-3p (1.49-fold, *p* < 0.001), and let-7b-3p (5.24-fold, *p* < 0.001) were found to be elevated in COVID-19 patients. Although a decrease in miR-16 was observed, it was not statistically significant (0.89-fold, *p* < 0.062). The expression levels of miR-590 (1.06-fold, *p* 0.815) showed no significant variation between COVID-19 patients and the control group. The differences in miRNA expression levels are shown in Figure 1.

**Figure 2:**
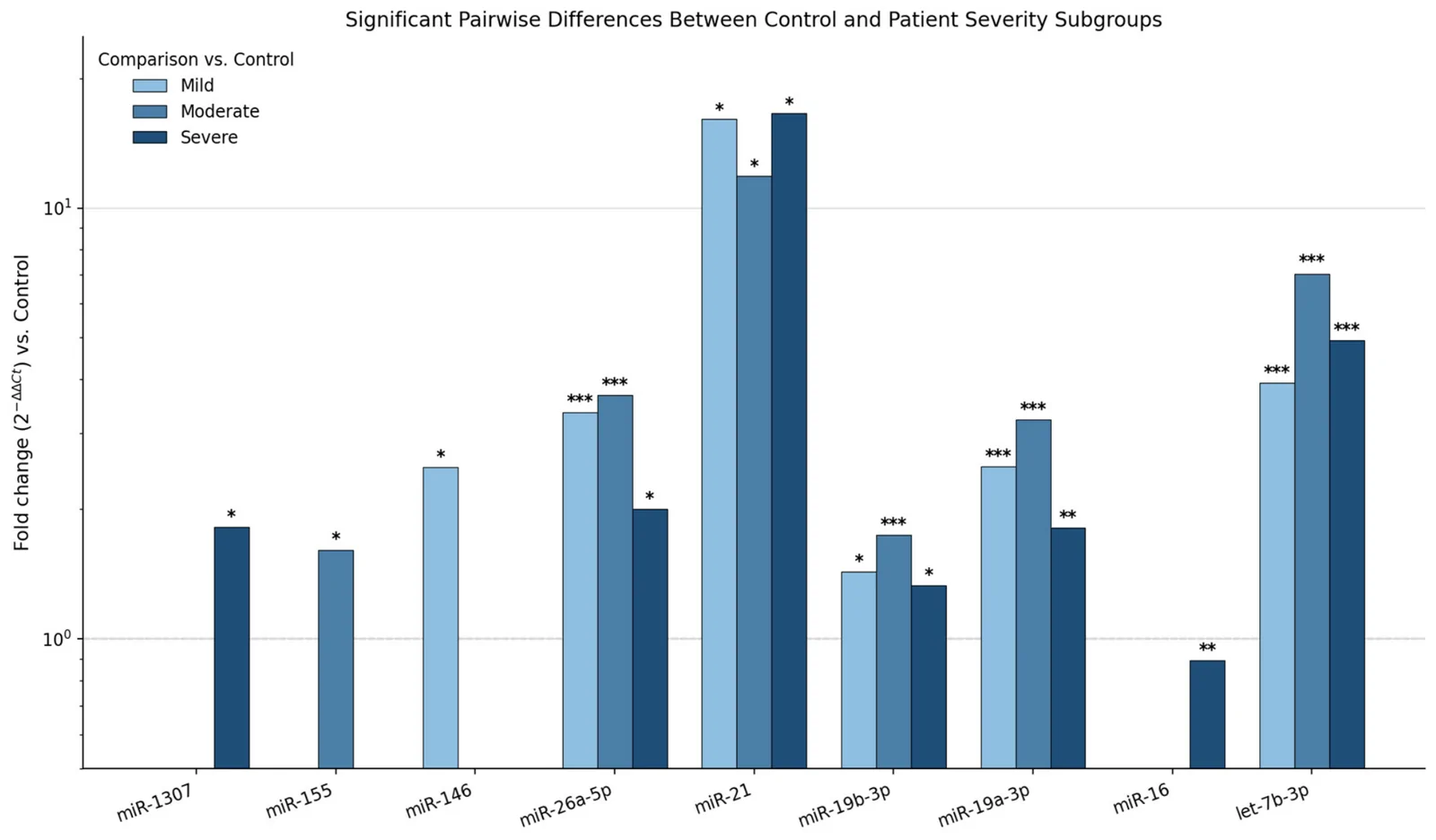
Fold-change (2^−TTct^) values for significant pairwise comparisons between control and each severity subgroup (mild, moderate, severe). Dashed line indicates no change (1.0×). * *p* < 0.05, ** *p* < 0.01, *** *p* < 0.001.

Subsequently, miRNA expression profiles of patients stratified by clinical severity (mild, moderate, and severe) were compared with the control group (Supplementary Table S2). Only pairwise comparisons that remained statistically significant after multiple-comparison correction (Benjamini–Hochberg for Kruskal–Wallis/Dunn, or Tukey HSD for one-way ANOVA, as applicable per miRNA) are shown in Figure 2. Comparisons that did not reach significance are ruled out. miR-26a-5p, miR-21, miR-19b-3p, miR-19a-3p, and let-7b-3p are significantly higher in all patient groups compared to controls. They distinguish the presence of the disease but not its clinical severity.

### Reference Gene Variability and Sensitivity Analysis

U6 Ct values showed a low overall coefficient of variation (2.35%) but differed significantly across the control, mild, moderate, and severe groups (Kruskal–Wallis, H = 41.11, *p* < 0.0001, ε^2^ = 0.42), with the largest difference observed in the moderate group (Supplementary Table S3). Raw target miRNA Ct values showed statistically significant positive correlations with U6 Ct values for six of the ten miRNAs (r = 0.22–0.40; Supplementary Table S4), indicating a degree of shared variation between U6 and the target assays. When all group comparisons were repeated after rank-based covariate adjustment for U6 Ct, the statistical significance and overall interpretation of the group effect remained unchanged for all ten miRNAs (Supplementary Table S5). These findings indicate that the principal between-group results were robust to explicit adjustment for the observed variation in U6 Ct.

The predicted inflammation-related target genes and affected pathways of the selected miRNAs are shown in Table 5. Bioinformatics analysis identified several potential inflammatory target genes of the selected miRNAs. STRING protein–protein interaction network analysis demonstrated a significantly interconnected network among these genes. Key hub genes included TLR4, TRAF6, IRAK1, PTEN, SOCS1, STAT3, MAPK1 and IL6, suggesting that these genes play central roles in inflammatory signaling. Functional enrichment analysis revealed significant involvement in major inflammatory pathways such as NF-κB signaling, Toll-like receptor (TLR) signaling, TNF signaling, PI3K-Akt signaling, MAPK signaling, and TGF-β signaling pathways.

## 4. Discussion

While circulating miRNAs are broadly implicated in host antiviral responses [24,25], the present study assessed expression differences rather than direct miRNA–viral genome interactions.

It should be noted that the pathway-level mechanisms discussed below are based on predicted miRNA–target gene interactions derived from bioinformatic analysis (TargetScan, miRDB, and miRTarBase, followed by STRING protein–protein interaction and KEGG pathway enrichment analysis; Table 5), supplemented by previously published functional studies cited throughout the text. As the present study measured miRNA concentrations in serum rather than intracellular compartments, these pathway associations, both predicted, and derived from the literature, should be regarded as biological context for the observed serum expression differences rather than as intracellular pathway activity directly demonstrated in this cohort.

A key finding of this study is the distinction between differences associated with disease presence and those associated with disease severity. While miR-26a-5p, miR-21, miR-19a-3p, miR-19b-3p, and let-7b-3p showed significant differences between COVID-19 patients and healthy controls, none of the ten miRNAs showed statistically significant differences across the mild, moderate, and severe disease groups after correction for multiple comparisons. These findings suggest that the observed miRNA expression differences may be more closely associated with the presence of COVID-19 than with disease severity; however, their diagnostic or prognostic performance cannot be established without dedicated diagnostic accuracy analyses and independent validation.

In our study, a statistically significant difference in miR-1307 expression levels was identified between COVID-19 patients and the control group (*p* = 0.014; Figure 1, Table 3). A significant difference was also observed among the control, mild, moderate, and severe subgroups when analyzed separately (*p* = 0.020; Table 4). Notably, miR-1307 expression was upregulated by approximately 1.5-fold in COVID-19 patients relative to healthy controls. miR-1307-3p and miR-1307 have been previously recognized as lung-associated miRNAs, with miR-1307, in particular, identified as a critical regulatory element in pulmonary development, especially during the neonatal period [26]. Moreover, multiple miRNAs, including miR-1307-3p, have been implicated in modulating inflammatory pathways, notably those governed by TGF-β [27]. The TGF-β/SMAD signaling pathway, in which SMAD4 acts as a central mediator, has been implicated in the pathogenesis of COVID-19. Increased TGF-β activity has been associated with inflammation, immune dysregulation, and pulmonary fibrosis in, suggesting that dysregulation of this pathway may contribute to disease severity [28].

miR-155, a pivotal regulator across diverse viral infections, orchestrates the modulation of numerous biological processes. It exerts primary control over the JAK-STAT, TLR, and NF-κB signaling cascades and is intricately involved in both viral replication and the host antiviral immune response [9]. miR-155 has been shown to directly target TAB2, an adaptor protein involved in Toll-like receptor and interleukin-1 signaling pathways, thereby modulating NF-κB activation and inflammatory responses [29]. A study conducted on COVID-19 patients reported an upregulation of serum miR-155 levels [30]. Conversely, another study observed a decrease in serum miR-155 in COVID-19 patients, suggesting a potential association with the clinical course of the disease [31]. Although the difference in expression levels between the patient and control groups in our study was minimal (1.18-fold), we observed a decrease in miR-155 levels specifically in the severe patient group. This study features a larger patient group relative to numerous prior investigations on miR-155 and substantiates the decline in serum miR-155 levels observed in severe COVID-19 cases. Discrepancies in reported miR-155 levels within the literature may stem from variability in the timing of sample acquisition during different phases of the disease course.

miR-146a, a key regulator of inflammatory processes, ranks among the earliest miRNAs upregulated by antiviral immune responses. Moreover, recent studies suggest that miR-146a possesses the capacity to directly target the SARS-CoV-2 genome [24]. This miRNA plays a critical role in the negative regulation of proinflammatory cytokine production, thereby modulating the intensity of the inflammatory response [32,33]. Additionally, miR-146a targets TRAF6, reducing nitric oxide (NO) production in infected macrophages, and through this mechanism, it attenuates intracellular host defense [10].

A study involving pregnant women demonstrated an upregulation of miR-146 levels in COVID-19 patients. This increase is thought to contribute to the control of severe COVID-19 by limiting inflammatory tissue damage and negatively regulating NK cell-mediated cytotoxicity [33]. In a study involving eighteen COVID-19 patients and fifteen healthy individuals, miR-146 expression levels were reported to be 2.8-fold higher in the patient group compared to the healthy controls [18]. Similarly, another study reported a 2.3-fold increase in miR-146 expression in patients with mild COVID-19 and a 1.9-fold increase in those with severe disease compared to healthy participants [31]. In our study, serum miR-146 levels exhibited a 2.2-fold increase in the patient group compared to controls. This elevation may be attributed to the immunomodulatory role of miR-146 in dampening inflammatory responses. Additionally, it is conceivable that SARS-CoV-2 promotes the upregulation of miR-146 as a potential mechanism to evade host immune surveillance.

Although miR-26 is not exclusively cardiac-specific, it is predominantly expressed within the cardiovascular system. miR-26a-5p plays a pivotal role in the biology of cardiomyocytes and is implicated in various pathophysiological processes such as cardiac hypertrophy, oxidative stress, and atrial fibrillation [12]. miR-26a has been reported to inhibit TGF-β-induced epithelial–mesenchymal transition by targeting SMAD4 and reducing fibrosis-related gene expression [34]. In our study, serum miR-26a-5p levels exhibited an approximately 2.8-fold increase in COVID-19 patients. Considering the established cardioprotective properties of miR-26a-5p, its upregulation in the context of COVID-19 may represent an adaptive response aimed at preserving cardiac function or mitigating pulmonary fibrosis. In addition, it is well established that cardiac complications are frequently observed in patients with severe COVID-19. In our study, the detection of lower miR-26a-5p levels in the severe patient group compared to other groups further supports this association.

The literature contains a limited number of studies examining miR-26a-5p in COVID-19 patients. Notably, a study by Park et al. identified 17 host miRNAs, including miR-26a-5p, with the capacity to interact with the SARS-CoV-2 genome. Among these, miR-26a-5p, miR-23a-3p, miR-103a-3p, and miR-92a-3p were identified as the most significantly upregulated miRNAs. These miRNAs are hypothesized to be involved in the suppression of proinflammatory responses and the inhibition of viral replication [35]. A study analyzing post-mortem lung biopsies from COVID-19 patients demonstrated a downregulation of miR-26a-5p in lung tissues relative to controls [36]. We believe that our investigation helps bridge this critical gap in the existing literature.

In the context of COVID-19, miR-21-3p has been identified as the miRNA with the propensity for pronounced upregulation in murine lung tissue [37]. miR-21 regulates inflammatory signaling by targeting PDCD4, a key modulator of NF-κB signaling, thereby influencing cytokine production and immune responses [14]. In a clinical study, the upregulation of miR-21 was suggested to serve as an indicator of chronic myocardial injury and inflammation [30]. In a study comprising six patients with severe and six with moderate COVID-19, reduced levels of miR-146a-5p and miR-21-5p were proposed as potential biomarkers indicative of severe disease manifestation [38]. In our study, serum miR-21 levels demonstrated a 14.6-fold elevation, underscoring its potential utility as a diagnostic biomarker in SARS-CoV-2 infection. In severe COVID-19 patients, the upregulation of serum miR-21 may be indicative of cardiac injury, while in the mild disease group, it might reflect T-cell activation.

The increased expression of miR-21 observed in COVID-19 patients may be associated with the activation of key inflammatory signaling pathways, including the PI3K–Akt and NF-κB pathways. Activation of these pathways may promote pulmonary inflammation, increased cytokine production, and tissue remodeling processes that contribute to lung injury and fibrosis during SARS-CoV-2 infection. Therefore, elevated miR-21 levels may reflect both inflammatory activation and the progression of immune-mediated tissue damage in COVID-19, underscoring its potential utility as a potential biomarker for COVID-19.

miR-19 exerts critical functional mechanisms in the development and disease processes of cardiac, vascular, and neuronal tissues. miR-19b-3p is considered a key regulator of the NF-κB signaling pathway via TNFAIP3 and may play a role in inflammation, immune response, and the pathogenesis of various diseases [15]. In a study involving 33 COVID-19 patients, plasma miR-19a-3p, miR-19b-3p, and miR-92a-3p were highlighted as potential biomarkers for the diagnosis of SARS-CoV-2 infection [20]. Increased expression of miR-19a-3p and miR-19b-3p may contribute to the dysregulation of inflammatory signaling pathways in COVID-19. These miRNAs are known to target PTEN and TNFAIP3, leading to activation of the PI3K/Akt and NF-κB signaling pathways, respectively. This activation may enhance immune cell function and promote the production of pro-inflammatory cytokine. In the present study, elevated serum levels of miR-19a-3p (2.44-fold) and miR-19b-3p (1.49-fold) were identified in COVID-19 patients. Despite intergroup variability limiting their utility for disease stratification, we suggest that these miRNAs hold promise as potential biomarkers for COVID-19 diagnosis.

Although this change did not reach statistical significance, miR-16 regulates cell survival and inflammatory responses by targeting genes such as BCL2 and TLR4. The suppression of BCL2 by miR-16 reduces anti-apoptotic signaling and promotes mitochondrial apoptosis, facilitating the elimination of infected or damaged cells [16]. Additionally, miR-16 can modulate TLR4 expression, thereby influencing Toll-like receptor-mediated inflammatory signaling pathways [39].

In the analysis conducted by Eyileten et al., miR-16 expression was not directly associated with mortality in COVID-19 patients. However, its levels were reduced in individuals with prolonged hospital stays [40]. Similarly, De Gonzalo-Calvo et al. reported that in blood samples obtained from 36 COVID-19 patients admitted to the intensive care unit and 43 patients hospitalized in general wards, miR-16 expression levels were elevated in ward patients, whereas markedly reduced levels were observed in those requiring intensive care [19].

In this study, miR-16 demonstrated decreased expression levels in COVID-19 patients relative to the control group. The decreased expression of miR-16 may lead to the increased expression of its target genes such as BCL2 and TLR4, resulting in inhibited apoptosis and enhanced inflammatory signaling. Such a profile may reflect a compromised anti-inflammatory response associated with SARS-CoV-2 infection. Nevertheless, notable variations in miR-16 expression were observed across different patient subgroups. As a result, it was concluded that miR-16 may not serve as a reliable biomarker for assessing disease severity.

Furthermore, miRNAs of the let-7 family, including let-7b-3p, play an important regulatory role in innate immune signaling and inflammatory responses. These miRNAs can modulate the expression of key inflammatory genes such as TLR4 and IL6, thereby influencing Toll-like receptor signaling and cytokine production [17]. A bioinformatics analysis confirmed that let-7 suppresses SARS-CoV-2 replication by targeting the S and M proteins. The same study also demonstrated that let-7 can inhibit the expression of multiple inflammatory factors by downregulating various cytokines and chemokines, including IL-1β, IL-6, IL-8, TNF-α, and VEGF-α [17].

Donyavi et al. reported a significantly higher expression level of let-7b-3p during the acute phase of COVID-19 compared to the convalescent phase [18]. In our study, let-7b-3p expression levels in COVID-19 patients exhibited a 5.2-fold increase. This miRNA may be a diagnostic marker for COVID-19. Although differences in expression were observed among patient groups, these variations were not statistically significant. This investigation stands among the few studies documenting an upregulation of let-7b-3p in COVID-19 patients. Let-7b-3p is postulated to play a significant role in attenuating both inflammatory processes and viral replication. However, further research is warranted to elucidate these mechanisms.

## 5. Conclusions

Although there are studies in the literature highlighting the role of miRNAs in viral infections, there remains a need for clinical research specifically focusing on host miRNAs expressed during the pandemic SARS-CoV-2 infection. A portion of the existing studies is based on bioinformatic analyses or animal models. This study was conducted with the aim of contributing to the identification of potential miRNAs that modulate the immune response against SARS-CoV-2 and to elucidate the mechanisms underlying the clinical manifestations caused by the virus—particularly by focusing on aberrant miRNA expression patterns involved in the COVID-19-related inflammatory response.

In our study, we observed a pronounced upregulation in the serum expression levels of miR-1307-3p, miR-146, miR-26a-5p, miR-21, miR-19a-3p, miR-19b-3p, and let-7b-3p in patients with COVID-19. No significant alterations were detected in the expression levels of miR-590 and miR-16. However, miR-155 expression was decreased in patients with severe COVID-19. While variations in miRNA expression were observed among patient groups, these differences did not reach statistical significance. Certain circulating miRNAs (miR-26a-5p, miR-21, miR-19a-3p, miR-19b-3p, let-7b-3p) can distinguish the presence of SARS-CoV-2 infection from healthy individuals; however, these miRNAs do not provide a reliable distinction between degrees of disease severity.

Despite reports of these miRNAs in diverse pathological conditions, there is a paucity of research clarifying their roles in viral infections. Consequently, it is imperative that their involvement in COVID-19 be explored through more extensive studies. miR-21 and let7b-3p may be potential biomarkers. Within the panel of miRNAs analyzed in our study, miR-26a-5p is distinguished by its cardioprotective properties, while miR-21 is particularly noteworthy for its correlation with cardiac injury. Increased expression of Let-7b-3p can suppress excessive inflammatory signaling and regulate cytokine production, thus potentially contributing to the control of inflammatory responses during viral conditions. The observed expression pattern suggests the simultaneous activation of pro-inflammatory and regulatory pathways in COVID-19. The upregulation of miR-19a/b and miR-21 together with the downregulation of miR-16 may promote inflammatory signaling through the PI3K-Akt and NF-κB pathways, whereas increased levels of miR-146a and let-7b may represent compensatory mechanisms aimed at limiting excessive inflammation. These pathway-level interpretations are based on predicted miRNA–target gene interactions derived from bioinformatic analysis (Table 5) and the prior literature, rather than on direct quantification of circulating cytokines or downstream signaling proteins in this cohort; they should therefore be regarded as hypothesis-generating rather than confirmatory.

### Limitation

A limitation of this study is the significant age and gender imbalance among disease severity groups, a characteristic inherent in COVID-19 epidemiology during the study period: older and male patients were disproportionately represented among moderate and severe cases (control range 25–53 years vs. moderate/severe range 49–88 years). To address this, a rank-based analysis of covariance (Quade’s method) was performed for each miRNA, and the principal findings—miR-26a-5p, miR-21, miR-19a-3p, miR-19b-3p, miR-16, and let-7b-3p—remained statistically significant after age adjustment, supporting that these associations are not fully attributable to the age imbalance between groups. A quadratic age term was additionally tested to evaluate potential non-linear effects of aging on miRNA expression; this term was not statistically significant for any of the ten miRNAs (all *p* > 0.18), and group-effect estimates were materially unchanged after its inclusion, providing no evidence of non-linear age confounding. An age-matched subgroup sensitivity analysis was considered but was not statistically feasible, as only 2–5 individuals per group fell within the narrow overlapping age band (45–55 years) between the control and moderate/severe groups. For miR-1307-3p and miR-146a, statistical significance was attenuated—though not lost—under age adjustment, and these findings should therefore be interpreted with greater caution than the other significant miRNAs. Given the near-complete absence of age overlap between the control and moderate/severe groups, residual confounding by age, sex, and unmeasured comorbidities cannot be entirely ruled out despite these adjustments. Other limitations of the study include its small sample size and single-center nature; future studies employing age- and sex-matched recruitment are warranted to confirm these associations independently of demographic confounders. Vaccination status was inconsistently recorded across the cohort (available for only 28% of patients, with uneven distribution across severity groups) and could therefore not be reliably analyzed as a covariate. Sample processing was not randomized across disease-severity groups, which may have introduced systematic processing-related variation. Although sensitivity analyses indicated that the principal findings were robust to adjustment for the observed U6 variability (see Results), residual technical confounding cannot be completely excluded. Future studies should therefore employ randomized, group-interspersed sample processing and prospectively validate candidate endogenous reference genes.

## Figures and Tables

**Table 1:** Baseline demographic and clinical characteristics. Age and sex (all groups, including control).

Variable	Control (n = 30)	Mild (n = 20)	Moderate (n = 25)	Severe (n = 25)	*p*
Age, years ^a^	36.0 ± 8.9	40.05 ± 18.33	65.60 ± 8.13	66.88 ± 11.72	<0.001
Sex ^c^					0.003
Male, n (%)	15 (50.0%)	5 (25.0%)	17 (68.0%)	19 (76.0%)	
Female, n (%)	15 (50.0%)	15 (75.0%)	8 (32.0%)	6 (24.0%)	

^a^ One-way ANOVA (Mean±SD); ^c^ Pearson’s Chi-squared test.

**Table 2:** Baseline demographic and clinical characteristics. clinical and biochemical parameters (patient severity subgroups only).

Variable	Mild (n = 20)	Moderate (n = 25)	Severe (n = 25)	*p*
CRP (mg/L) ^a^	9.06 ± 8.53	141.43 ± 77.50	180.92 ± 81.68	<0.001
LDH (U/L) ^b^	192.00 (164.25–215.25)	331.00 (269.00–402.00)	416.00 (333.00–636.00)	0.001
Ferritin (µg/L) ^b^	61.80 (33.62–185.00)	465.00 (255.00–1179.00)	994.00 (504.00–2584.00)	<0.001
WBC (10^3^/µL) ^a^	6.92 ± 2.93	6.47 ± 2.87	7.83 ± 6.01	0.5
Neutrophils (10^3^/µL) ^a^	4.67 ± 2.98	5.08 ± 2.60	6.64 ± 4.89	0.2
Lymphocytes (10^3^/µL) ^b^	1.39 (1.24–1.65)	0.85 (0.55–1.19)	0.50 (0.29–0.86)	0.024
Platelets (10^3^/µL) ^a^	246,150 ± 82,374	186,400 ± 68,552	198,200 ± 131,418	0.12
D-dimer (ng/mL) ^b^	350.0 (200.0–525.0)	401.0 (282.0–1167.0)	2400.0 (900.0–4000.0)	<0.001
Fibrinogen (mg/dL) ^a^	324.60 ± 58.71	557.64 ± 233.26	516.96 ± 155.39	<0.001
Length of hospital stay, days ^a,d^	Outpatient (N/A)	7.76 ± 1.45	20.84 ± 5.36	<0.001

CRP, C-reactive protein; LDH, lactic acid dehydrogenase; WBC, white blood count. ^a^ One-way ANOVA (Mean ± SD); ^b^ Kruskal–Wallis rank sum test (median (25th–75th percentile)); ^d^ Welch’s *t*-test.

**Table 3:** Comparison of miRNA values of groups.

miRNA	Control (n = 30)	COVID-19 Patients (n = 70)	*p*
miR-1307 ^b,c^	11.76 (11.31–12.46)	11.34 (10.89–11.78)	0.014
miR-155 ^b,c^	16.02 (15.45–16.97)	15.71 (14.73–16.45)	0.147
miR-146 ^a,d^	13.28 ± 2.18	12.14 ± 1.21	0.011
miR-26a-5p ^b,c^	15.31 (14.72–15.87)	13.44 (12.49–14.63)	<0.001
miR-21 ^b,c^	17.63 (15.85–24.55)	14.96 (14.15–16.62)	<0.001
miR-19b-3p ^b,c^	12.58 (12.05–13.04)	11.36 (10.55–11.92)	<0.001
miR-19a-3p ^b,c^	12.20 (11.70–12.72)	10.77 (9.78–11.13)	<0.001
miR-16 ^b,c^	10.29 (9.77–10.89)	10.97 (9.79–11.48)	0.063
miR-590 ^a,e^	13.94 ± 1.70	13.85 ± 1.66	0.815
let-7b-3p ^b,c^	16.52 (15.85–17.05)	14.13 (13.39–15.38)	<0.001

^a^ Mean ± SD, ^b^ Median (25th–75th percentile), ^c^ Mann–Whitney U, ^d^ Welch *t*-test, ^e^ Student’s *t*-test.

**Table 4:** Comparison of control and patient groups.

	Control (n = 30 ^1^)	Mild (n = 20 ^1^)	Moderate (n = 25 ^1^)	Severe (n = 25 ^1^)	*p* ^2^	*p* ^3^
miR-1307 ^a^	11.76 (11.31–12.46)	11.28 (10.91–11.86)	11.67 (11.28–12.09)	11.08 (10.82–11.51)	0.020	0.047
miR-155 ^a^	16.02 (15.45–16.97)	15.56 (14.55–15.92)	15.02 (14.36–15.72)	16.41 (15.88–17.26)	0.2	0.075
miR-146 ^1^	13.28 ± 2.18	11.96 ± 1.49	12.19 ± 1.14	12.24 ± 1.06	0.013	0.031
miR-26a-5p ^a^	15.31 (14.72–15.87)	13.04 (12.33–14.29)	13.12 (12.01–14.66)	13.63 (13.07–14.70)	<0.001	<0.001
miR-21 ^a^	17.63 (15.85–24.55)	14.83 (14.07–16.53)	14.99 (14.37–16.83)	14.94 (13.89–16.04)	<0.001	<0.001
miR-19b-3p ^a,4^	12.58 (12.05–13.04)	11.59 (10.87–11.74)	11.01 (10.44–11.68)	11.58 (10.66–12.01)	<0.001	0.003 ^5^
miR-19a-3p ^a^	12.20 (11.70–12.72)	10.61 (9.69–11.01)	10.00 (9.06–11.05)	10.98 (10.40–11.47)	<0.001	<0.001
miR-16 ^a^	10.29 (9.77–10.89)	11.04 (10.28–11.46)	9.78 (9.35–10.60)	11.29 (11.10–12.08)	0.6	0.528
miR-590 ^1^	13.94 ± 1.70	13.63 ± 1.52	14.37 ± 1.49	13.52 ± 1.87	0.3	0.250
Let-7b-3p ^a^	16.52 (15.85–17.05)	14.47 (13.92–15.80)	13.74 (12.98–15.04)	14.00 (13.79–15.12)	<0.001	<0.001

^1^ Mean ± SD; n (%); ^a^ Median (25th–75th percentile); ^2^ One-way ANOVA; ^3^ ANCOVA; ^4^ Kruskal–Wallis; ^5^ Ranke base-ANCOVA (Quade’s method).

**Table 5:** Predicted inflammation-related target genes of the selected miRNAs and their associated signaling pathways.

miRNA	Target Gene	Associated Inflammatory Pathway	Functional Role
miR-1307	SMAD4	TGF-β signaling pathway	Regulation of inflammatory signaling
miR-155	TAB2	NF-κB signaling pathway	Adaptor protein involved in TLR signaling
miR-146a	TRAF6/IRAK1	Toll-like receptor signaling pathway	Activation of NF-κB signaling/Mediates innate immune signaling
miR-21	PTEN	PI3K–Akt signaling pathway	Regulation of inflammation and cell survival
miR-21	PDCD4	NF-κB signaling pathway	Regulation of inflammatory gene expression
miR-26a-5p	SMAD1/SMAD4	TGF-β signaling pathway	Immune regulation/Transcriptional mediator in TGF-β signaling
miR-19a-3p	PTEN	PI3K–Akt signaling pathway	Regulation of inflammatory responses
miR-19b-3p	TNFAIP3	NF-κB signaling pathway	Negative regulator of inflammation
miR-16	BCL2	Apoptosis-related pathways	Regulation of cell survival
miR-16	TLR4	Innate immune signaling pathway	Lipopolysaccharide (LPS) receptor
miR-590	TGFBR2	TGF-β signaling pathway	Immune modulation
let-7b-3p	TLR4	Toll-like receptor signaling pathway	Regulation of innate immune response
let-7b-3p	IL6	Cytokine signaling pathway	Pro-inflammatory cytokine

The table summarizes the predicted target genes of the selected miRNAs and their involvement in major inflammatory signaling pathways, including NF-κB, Toll-like receptor, PI3K–Akt, and TGF-β signaling pathways. SMAD4: SMAD Family Member 4, TAB2: TGF-β Activated Kinase 1 Binding Protein 2, TRAF6: TNF Receptor Associated Factor 6, IRAK1: Interleukin-1 Receptor Associated Kinase 1, PTEN: Phosphatase and Tensin Homolog, PDCD4: Programmed Cell Death 4, SMAD1: SMAD Family Member 1, TNFAIP3: Tumor Necrosis Factor Alpha-Induced Protein 3, BCL2: B-Cell Lymphoma 2, TLR4: Toll-Like Receptor 4, TGFBR2: Transforming Growth Factor Beta Receptor 2, IL6: Interleukin-6.

## Data Availability

The data presented in this study are available on request from the corresponding author. The data are not publicly available due to privacy or ethical restrictions.

## Supplementary Materials

The following supporting information can be downloaded at: https://www.mdpi.com/article/10.3390/microorganisms14092064/s1, Table S1: Linear vs. quadratic age-adjustment models (ANCOVA); Table S2: Relative expression (fold-change) values; Table S3: U6 Ct values were compared across the control, mild, moderate, and severe groups; Table S4: Pearson correlation between U6 Ct and raw target miRNA Ct; Table S5: Comparison of established (unadjusted) and U6-covariate-adjusted group-effect *p*-values for all ten miRNAs.

## Abbreviations

The following abbreviations are used in this manuscript:

miRNA	microRNA
PCR	polymerase chain reaction
cDNA	Complementary DNA
Ct	cycle threshold
