# Supplementary material for: Expression Analysis of Selected miRNAs in COVID-19 Patients
Source: Microorganisms. 2026 Sep 16;14(9):2064. doi: 10.3390/microorganisms14092064 (PMC13609553; doi:10.3390/microorganisms14092064)
Supplement: Supplementary file 1 [file microorganisms-14-02064-s001.zip › microorganisms-4549195-supplementary.pdf]

Supplementary Tables

Supplementary Table S1. Linear vs. Quadratic Age-Adjustment Models (ANCOVA)

Comparison of group-effect p-values from linear (age-only covariate) versus quadratic (age + age^2 covariates) ANCOVA models for each miRNA, testing for potential non-linear effects of age.

miRNA	Linear ANCOVA p (group effect)	Quadratic ANCOVA p (group effect)	age^2 term p
**miR-1307**	0.047	0.061	0.240
**miR-155**	0.075	0.070	0.768
**miR-146**	0.031	0.033	0.840
**miR-26a-5p**	<0.001	<0.001	0.386
**miR-21**	<0.001	<0.001	0.737
**miR-19b-3p**	0.226	0.234	0.730
**miR-19a-3p**	<0.001	<0.001	0.469
**miR-16**	0.528	0.593	0.520
**miR-590**	0.250	0.350	0.184
**let-7b-3p**	<0.001	<0.001	0.462

Supplementary Table S1. "age^2 term p" indicates whether the quadratic term itself was statistically significant.

Supplementary Table S2. Relative Expression (Fold-Change) Values

miRNA	Control	Mild	Moderate	Severe
**miRNA-1307**[a]	1.00x	1.47x	1.34x	1.82x
**miRNA155**[a]	1.00x	1.31x	1.61x	0.80x
**miRNA-146**[b]	1.00x	2.50x	2.13x	2.05x
**miRNA26-5p**[a]	1.00x	3.36x	3.68x	2.00x
**miRNA21-3p**[a]	1.00x	16.11x	11.85x	16.62x
**miRNA-19b-3p**[a]	1.00x	1.43x	1.74x	1.33x
**miRNA19a-3p**[a]	1.00x	2.51x	3.23x	1.81x
**miRNA16**[a]	1.00x	0.67x	1.12x	0.89x
**miRNA590**[b]	1.00x	1.24x	0.74x	1.34x
**miRNA-let7b-3p**[a]	1.00x	3.93x	7.03x	4.93x

[a] Kruskal-Wallis; [b] One-way ANOVA.

Supplementary Table S3. U6 Ct values were compared across the Control, Mild, Moderate, and Severe groups

Group	n	Mean U6 Ct	SD	CV%
**Control**	30	19.139	0.355	1.86%
**Mild**	20	18.897	0.173	0.92%
**Moderate**	25	18.359	0.412	2.25%
**Severe**	25	18.866	0.322	1.71%

Supplementary Table S4. Pearson correlation between U6 Ct and raw target miRNA Ct

miRNA	Pearson r (U6 vs. raw target Ct)	p
**miR-1307**	0.331	**<0.001**
**miR-155**	0.129	0.1996
**miR-146**	0.252	**0.0114**
**miR-26a-5p**	0.284	**0.0042**
**miR-21**	0.168	0.0950
**miR-19b-3p**	0.118	0.2407
**miR-19a-3p**	0.219	**0.0283**
**miR-16**	0.291	**0.0034**
**miR-590**	0.124	0.2181
**let-7b-3p**	0.402	**<0.001**

Supplementary Table S5. Comparison of established (unadjusted) and U6-covariate-adjusted group-effect p-values for all ten miRNAs

miRNA	Established test p (KW/ANOVA, no U6 adjustment)	Quade rank U6-adjusted p	Result
**miR-1307**	**0.0096**	**0.0029**	Unchanged
**miR-155**	**0.0030**	**0.0020**	Unchanged
**miR-146**	**0.0131**	**0.0087**	Unchanged
**miR-26a-5p**	**<0.001**	**<0.001**	Unchanged
**miR-21**	**0.0062**	**0.0046**	Unchanged
**miR-19b-3p**	**<0.001**	**<0.001**	Unchanged
**miR-19a-3p**	**<0.001**	**<0.001**	Unchanged
**miR-16**	**<0.001**	**<0.001**	Unchanged
**miR-590**	0.2888	0.4130	Unchanged
**let-7b-3p**	**<0.001**	**<0.001**	Unchanged
